# Efficacy and safety of sodium bromfenac eye drops in the treatment of postoperative inflammation in cataract surgery: A protocol for systematic review and meta-analysis

**DOI:** 10.1097/MD.0000000000023131

**Published:** 2020-12-04

**Authors:** Chunyue Wang, Yana Cao, Xi Chen, Mingming Cai, Wei Huang

**Affiliations:** a Department of Ophthalmology, The Ninth People's Hospital of Chongqing, Beibei; b Department of Ophthalmology, Chungking General Hospital, Yubei; c Department of Ophthalmology, Chongqing Aier Mega Eye Hospital, Chongqing, China.

**Keywords:** cataract surgery, protocol, randomized controlled trials, sodium bromfenac, systematic review

## Abstract

**Background:**

A cataract is a degenerative change in the optical quality of the lens caused by protein denaturation. Modern medicine is mainly based on surgical treatment. Cataract surgery is often accompanied by severe inflammation, and glucocorticoid therapy has many adverse reactions and side effects. The non-steroidal anti-inflammatory drug sodium bromfenac not only has good anti-inflammatory, analgesic and anti-allergic effects, but also does not produce side effects caused by hormone drugs. Clinical studies have shown that sodium bromfenac eye drops have a good curative effect in treating postoperative inflammation of cataract, with low recurrence rate and certain therapeutic advantages, but lack of evidence-based medicine evidence. The purpose of this study is to systematically evaluate the efficacy and safety of sodium bromfenac eye drops in the treatment of postoperative inflammation of cataracts.

**Methods:**

Use computer to search English and Chinese databases, such as PubMed, Embase, Web of Science, the Cochrane Library, CNKI, Wanfang, Weipu, China Biomedical Database, and Chinese Clinical Trial Registry for randomized controlled trials on the treatment of postoperative postoperative inflammation in cataract surgery with sodium bromfenac eye drops from the establishment of the database to September 2020, and data extraction and literature quality evaluation were conducted independently by two researchers, and Meta analysis was conducted on the included literature using RevMan5.3 software.

**Results:**

In this study, the efficacy and safety of sodium bromfenac eye drops in the treatment of postoperative inflammation of cataract surgery were evaluated by the effective rate, symptom score, adverse reactions, incidence, recurrence rate, etc.

**Conclusion:**

This study will provide reliable evidence-based evidence for the clinical application of bromofenac sodium eye drops in the treatment of postoperative inflammation of cataract.

**OSF Registration number:**

DOI 10.17605/OSF.IO/3KP7R

## 1 Introduction

Cataract is a blinding disease caused by the degeneration of lens protein. Phacoemulsification combined with intraocular lens implantation is the international standard surgical method for cataract, and has been popularized and applied worldwide.^[1]^ However, cataract surgery is often accompanied by serious complications, such as endophthalmitis, corneal injury, macular cystoid edema, and secondary glaucoma,^[2]^ which greatly affect the surgical effect and visual quality of patients. Endophthalmitis is one of the most serious complications of cataract surgery, the incidence rate in small and medium-sized ophthalmic institutions in China is 0.11%,^[3]^ although the incidence is low, but because of the complicated environment of eye, bacterial infections and severe inflammation, it is destructive and lead to a rapid progression once happened, making it a serious threat to vision, and even lead to irreversible blindness.^[4]^ Glucocorticoids inhibit phospholipase A2 from producing arachidonic acid, thus reducing the production of prostaglandin and leukotriene. It has strong anti-inflammatory effect and is widely used in the treatment of postoperative inflammation in cataract surgery. But it has many adverse reactions and side effects.^[5]^

Non-steroidal anti-inflammatory drugs (NSAIDs) not only have good anti-inflammatory, analgesic and anti-allergic effects, but also do not produce clinical side effects like which caused by hormone anti-inflammatory drugs. As a new generation of NSAIDs, sodium bromfenac has strong anti-inflammatory and analgesic effect and is safe. Its penetration to the eye tissue is strong, and the drug concentration lasts long.^[6]^ Studies abroad have shown that after cataract surgery, sodium bromfenac prevent postoperative inflammation by inhibiting the production of PGE2, which is more effective and safe than glucocorticoids(GCs).^[7]^

At present, several randomized controlled studies^[7–9]^ have shown that sodium bromfenac eye drops can avoid unnecessary side effects of hormone therapy after cataract surgery, have good anti-inflammatory and analgesic effects, and significantly reduce the recurrence rate. However, there are differences in the research scheme and curative effect of each clinical trial, which leads to the uneven results, which to some extent affects the promotion of the therapy. Therefore, this study plan to systematically evaluate the efficacy and safety of sodium bromfenac eye drops in the treatment of postoperative inflammation in cataract surgery, and provided a reliable evidence-based basis for the clinical application of sodium bromfenac eye drops in the treatment of postoperative inflammation in cataract surgery.

## 2 Methods

### 2.1 Protocol register

This protocol of systematic review and meta-analysis has been drafted under the guidance of the preferred reporting items for systematic reviews and meta-analyses protocols (PRISMA-P). Moreover, it has been registered on open science framework (OSF) on October 3, 2020 (Registration number: DOI 10.17605/OSF.IO/3KP7R).

### 2.2 Ethics

Since this is a protocol with no patient recruitment and personal information collection, the approval of the ethics committee is not required.

### 2.3 Eligibility criteria

#### 2.3.1 Types of studies

We will collected all available randomized controlled trails (RCTs)on sodium bromfenac eye drops in the treatment of postoperative inflammation in cataract surgery, regardless of blinding, publication status, region, but Language will be restricted to Chinese and English.

#### 2.3.2 Research objects

Patients who aged ≥18 years old, underwent cataract surgery and complicated with inflammation, with no limitation to the type of cataract surgery. Patients with diabetes and autoimmune diseases were excluded. The patients’ nationality, race, sex, course of disease and so on are unlimited.

#### 2.3.3 Interventions

The treatment group was treated with bromfenac sodium eye drops and the control group was treated with placebo or other drugs. There was no limit to the type, dosage, frequency, course of treatment of placebo or other drugs.

#### 2.3.4 Outcome indicators

(1) Primary outcome: ocular inflammation score
(2) Secondary outcomes: visual acuity rating, pain, intraocular pressure, corneal central thickness

### 2.4 Exclusion criteria

(1) Duplicate published papers;
(2) Studies published as abstracts or with incomplete data and unable to obtain complete data after contacting the author;
(3) Studies with obvious errors in the data;
(4) Studies in which intervention combined with other therapies, such as laser, traditional Chinese medicine, acupuncture, auricular points, etc;
(5) Studies without relevant outcome indicators.

### 2.5 Search strategy

“xiu fen suan”(bromfenac), “xiu fen suan na”(bromfenac sodium), “bai nei zhang”(cataract), etc. were used as Chinese search terms and retrieved in Chinese database, including China Knowledge Network (CNKI), Wanfang Data Knowledge Service Platform, VIP Information Chinese Journal Service Platform (VIP), China Biomedical Database; and “bromfenac”, “bromfenac sodium”, “cataract”, etc. were used as English search terms and retrieved in English database, including PubMed, EMBASE, Web of Science, the Cochrane Library. The retrieval time was from the establishment of the database to September 2020. All domestic and foreign literatures on the treatment of postoperative inflammation after cataract surgery were collected. Take PubMed as an example, and the retrieval strategies are shown in Table 1.

**Table 1:** Search strategy in PubMed database.

Number	Search terms
#1	Bromfenac [Mesh]
#2	Bromfenac sodium [Title/Abstract]
#3	Bromophenol acid sodium [Title/Abstract]
#4	Sodium bromfenac[Title/Abstract]
#5	Sodium bromide Finn acid [Title/Abstract]
#6	#1 OR #2 OR #3 OR #4 OR #5
#7	Cataract [Mesh]
#8	Cataract surgery [Title/Abstract]
#9	#7 OR #8
#10	#6 AND #9

### 2.6 Data screening and extraction

**Figure 1:**
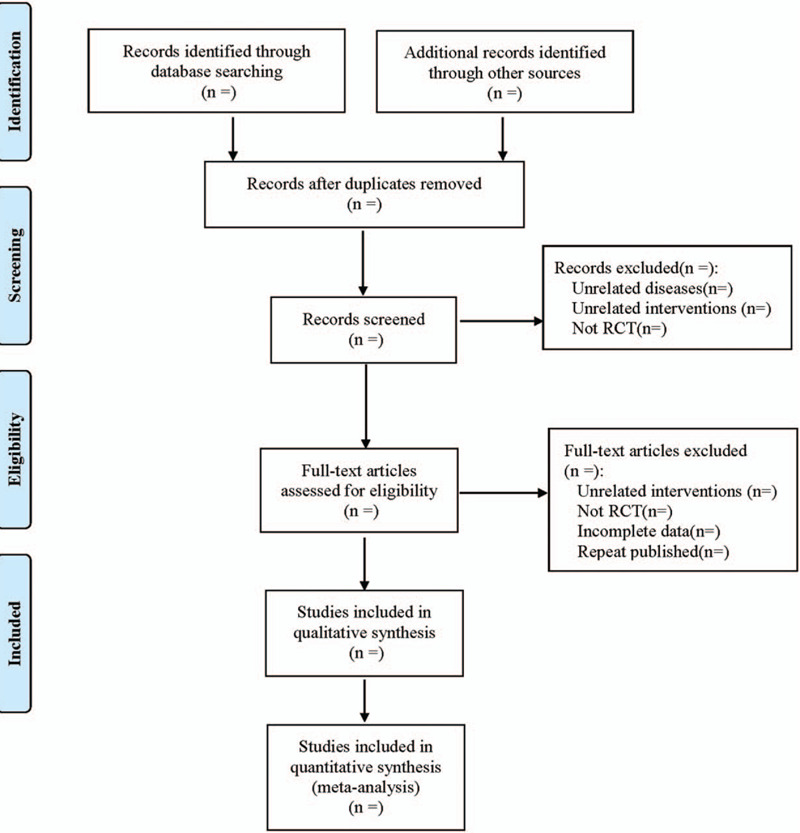
The process of literature filtering.

Referring to the method of research selection in version 5.0 of the Cochrane collaboration Network system Evaluator Manual, according to the PRISMA flow chart, the two researchers used the EndNote X9 document management software to independently screen and check the literature according to the above inclusion and exclusion criteria, and check each other, if there were different opinions, negotiate with a third party to resolve the differences. At the same time, Excel 2013 was used to extract relevant information, including: (1) Clinical study (title, first author, year of publication, sample size, sex ratio, mean age, mean course of disease, stage); (2) Intervention (dose, frequency, course of treatment of sodium bromfenac in treatment group; type, dose, frequency, course of treatment of placebo or other drugs in control group); (3) Risk bias assessment elements in randomized controlled trials; (4) Outcome indicators. The literature screening process is shown in Figure 1.

### 2.7 Literature quality assessment

Cochrane collaboration's tool for assessing risk of bias is used to assess the risk of bias for included studies. According to the performance of the literature included in the above evaluation items, the two researchers will give low risk, unclear or high risk judgments one by one, and cross-check each after completion. If there are differences, discussions will be held. If there is no agreement between the two, it will be discussed with third party researchers.

### 2.8 Statistical analysis

#### 2.8.1 Data analysis and processing

The RevMan 5.3 software provided by the Cochrane Collaboration will be used for statistical analysis. (1) relative risk (RR) is selected as the statistic for the dichotomous variable. For continuous variables, weighted mean difference (WMD) is selected when the tools and units of measurement indicators are the same, standardized mean difference (SMD) is selected with different tools or units of measurement, and all the above are represented by effect value and 95% confidence interval (CI). (2) Heterogeneity test: *I*^*2*^ value is used to quantitatively evaluate the inter-study heterogeneity. If *I*^2^ ≤ 50%, the heterogeneity is considered to be good, and the fixed-effect model is adopted. If *I*^2^ > 50%, it is considered to be significant heterogeneity, the source of heterogeneity will be explored through subgroup analysis or sensitivity analysis. If there is no obvious clinical or methodological heterogeneity, it will be considered as statistical heterogeneity, and the random-effect model will be used for analysis. At the same time, Stata14 is used for Egger's and Begg's test.

#### 2.8.2 Dealing with missing data

If there is missing data in the article, contact the author via email for additional information. If the author cannot be contacted, or the author has lost the relevant data, descriptive analysis will be carried out, not meta-analysis.

#### 2.8.3 Subgroup analysis

According to the types of cataract surgery, it can be divided into three subgroups: phacoemulsification, extracapsular cataract extraction and posterior chamber interocular lens implantation; According to the patient's age stage, it can be divided into three subgroups: young people (over 18 years old), middle-aged people and elderly people; Subgroup analysis according to the course of treatment.

#### 2.8.4 Sensitivity analysis

In order to test the stability of meta-analysis results of indicators and avoid low quality and small sample study to affect the results, a one-by-one elimination method will be adopted for sensitivity analysis.

#### 2.8.5 Assessment of reporting biases

Funnel plots were used to assess publication bias if no fewer than 10 studies were included in an outcome measure. Moreover, Egger's and Begg's test were used for the evaluation of potential publication bias.

#### 2.8.6 Evidence quality evaluation

The Grading of Recommendations Assessment, Development, and Evaluation (GRADE) will be used to assess the quality of evidence. It contains 5 domains (bias risk, consistency, directness, precision, and publication bias). And the quality of evidence will be rated as high, moderate, low, and very low.

## 3 Discussion

Cataract is a degenerative lesion caused by optical quality loss due to lens opacity.^[10]^ Studies have shown that inflammation after cataract surgery mainly results from mechanical injury in surgery, foreign body reaction of intraocular lens, and stimulation of membrane by residual lens epithelium,^[1]^ which activates phospholipase A2, releases arachidonic acid, and forms prostaglandin under the catalysis of cox-enzyme, resulting in various damages such as eye pain and allergy.^[11,12]^ Glucocorticoid drugs have strong anti-inflammatory effects and are widely used in the treatment of inflammation after cataract surgery. However, studies have shown that glucocorticoid can cause increased intraocular pressure and hormone-induced glaucoma, which is not accepted by some clinicians and patients.

In recent years, non-steroidal anti-inflammatory drug eye drops have been widely used in the prevention and treatment of inflammation after cataract surgery. Non-steroidal anti-inflammatory drug sodium bromfenac is a selective COX-2 inhibitor, which inhibits PGE2 by regulating COX-2 and has anti-inflammatory and analgesic effects several times as strong as other NSAIDs.^[13,14]^ As a lipophilic molecule that can penetrate into ocular tissue, bromfenac sodium can increase the duration of action in the eye and enhance clinical efficacy.^[15]^ Clinical studies have shown that sodium bromfenac can reduce surgery-induced pupil constriction, maintain intraoperative pupil dilation, inhibit anterior chamber inflammatory response, ease pain, etc. it has the advantages of not affecting wound healing, preventing high intraocular pressure, and less related adverse reactions compared with hormone drugs.^[16]^ It is believed that sodium bromfenac can be used as a reliable drug for complications of inflammation after cataract surgery.^[17,18]^

The clinical effect of sodium bromfenac eye drops on inflammation after cataract surgery is reliable. However, the evidence from RCTs is not consistent. With the increasing number of clinical trials, it is urgent to systematically evaluate the inflammation after cataract surgery with sodium bromfenac eye drops. In this study, we will summarize the latest evidence of the efficacy of sodium bromfenac eye drops in the treatment of inflammation after cataract surgery. This work also provides useful evidence for determining the efficacy and safety of sodium bromfenac eye drops in patients with postoperative inflammation after cataract surgery, which is beneficial for both clinical practice and health-related decision makers. However, this systematic review has some limitations. There may be some clinical heterogeneity in the treatment group with different sodium bromfenac dosages, and in the control group with different intervention methods. And due to the limitations of the original literature, no comparison was made with positive controls such as diclofenac sodium eye drops, pranoprofen eye drops, ketorolac trobutanol eye drops, and flurbiprofen sodium eye drops. Due to the limitation of language ability, we only search literature in English and Chinese, and may ignore research or reports in other languages.

## Author contributions

**Conceptualization:** Chunyue Wang.

**Data collection:** Xi Chen and Mingming Cai.

**Funding acquisition:** Chunyue Wang.

**Funding support:** Chunyue Wang.

**Literature retrieval:** Chunyue Wang and Yana Cao.

**Software operating:** Wei Huang.

**Software:** Wei Huang.

**Supervision:** Chunyue Wang.

**Writing – original draft:** Chunyue Wang and Yana Cao.

**Writing – review & editing:** Chunyue Wang, Yana Cao.
